# “They forget that I’m a human being”—ward round communication with older patients living with frailty and informal caregivers: a qualitative study

**DOI:** 10.1007/s41999-024-01043-5

**Published:** 2024-09-04

**Authors:** Lene Holst Andersen, Bo Løfgren, Mads Skipper, Kristian Krogh, Rune Dall Jensen

**Affiliations:** 1 Department of Internal Medicine, Randers Regional Hospital, Skovlyvej 15, 8930 Randers, NE Denmark; 2 Department of Geriatric Medicine, Aarhus University Hospital, Aarhus, Denmark; 3 Department of Clinical Medicine, Aarhus University, Aarhus, Denmark; 4 Postgraduate Medical Education, Region North, Viborg, Denmark; 5 Department of Anesthesiology and Intensive Care, Aarhus University Hospital, Aarhus, Denmark; 6 MidtSIM, Corporate HR, Central Denmark Region, Aarhus, Denmark

**Keywords:** Communication, Qualitative research, Patient-centred care, Family-centred care, Informal caregivers

## Abstract

**Purpose:**

Skilful communication prompts quality patient care. Informal caregivers occupy a crucial role when caring for hospitalised older patients living with frailty. However, skilful communication with both patients and informal caregivers during ward rounds has not been studied. Thus, we aimed to explore communication preferences of patients and informal caregivers during ward rounds.

**Methods:**

We conducted semi-structured interviews with hospitalized patients and informal caregivers until information redundancy occurred. We used inductive coding of the transcribed interviews followed by a reflexive thematic analysis.

**Results:**

The study included 15 patients and 15 informal caregivers. Patients had a median age of 85 years (range 75–100 years) and seven patients were females. Informal caregivers’ median age were 45 years (range 38–80 years) and 13 were females. Three themes were generated: (1) building relationships and conveying information, (2) alleviating informal caregiver strain and (3) sharing the decision-making process. Themes highlighted the importance of collaborative and empathetic approaches in healthcare interactions, emphasizing interpersonal communication skills, such as fostering professional relationships. The interviews unveiled informal caregiver burden stemming from disempowerment during hospital discharge process and managing mistrust within the healthcare system. The shared decision-making process should address patients’ and informal caregivers’ needs and circumstances.

**Conclusions:**

Communication preferences of a population of older patients living with frailty and informal caregivers during ward rounds encompass interpersonal communication, demonstrating ample time, and being seen as a human being. Informal caregivers value being included in the decision-making process. Skilful communication includes for doctors to recognize informal caregivers’ narratives and burdens.

**Graphical abstract:**

**Supplementary Information:**

The online version contains supplementary material available at 10.1007/s41999-024-01043-5.

## Key summary points

**Aim:**

To explore communication preferences of patients and informal caregivers during ward rounds.

**Findings:**

The study identified establishing professional relationships with patients and ensuring informal caregiver inclusion as the preferred communication preferences. Healthcare personnel should recognize informal caregiver burden and carefully dissect the shared decision-making process to ensure both patient and informal caregiver inclusion.

**Message:**

Healthcare personnel should recognize informal caregivers' burdens and ensure both patient and caregiver inclusion through empathetic and collaborative communication.

**Supplementary Information:**

The online version contains supplementary material available at 10.1007/s41999-024-01043-5.

## Graphical abstract

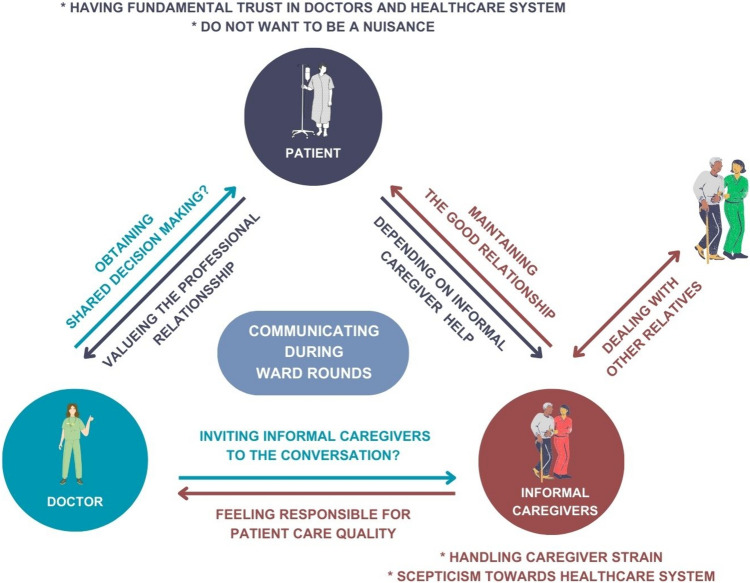

## Introduction

During a hospital stay, important patient care decisions are often made during ward rounds, and skilful communication is needed at these times [1]. Communication is best regarded as a dynamic process influenced by context and by all the individuals involved [2]. Rather than involving a linear transmission of messages, it reflects a complex interaction where people continuously send, receive and adjust messages and meanings [2]. The effectiveness of communication and of information exchange depends on the quality of interaction between participants, their mutual understanding and development of a holistic relationship [3]. It also requires that the individuals concerned can communicate. This may be more difficult for older hospital patients who are frail, with multiple comorbidities and functional decline [4] as well as are acutely unwell and perhaps suffering from conditions such as delirium [5].

Informal caregivers (ICs) are among the individuals who may be involved in ward round communication, as they typically participate during ward rounds or are contacted afterwards. They contribute significantly to patient care by providing valuable insights into patient preferences and assisting with patient discharge [6, 7]. This aligns with the family-centred care perspective, a holistic approach to patient care [8] that has been shown in studies to improve health outcomes and enhance the care experience for both patients and their families. [9, 10]. However, as previously stated, patients’ and ICs’ communication preferences may differ, which may make effective communication during ward rounds challenging [11]. As such, considering the communication preferences of both patients and ICs is essential to comprehensively understand the requisites for effective ward round communication, which may ultimately improve patient outcomes and patient safety. However, their communication preferences must be explored holistically instead of merely as verbal actions.

Therefore, in this study, we aimed to explore the communication preferences of older patients living with frailty and of their informal caregivers during the patients’ hospitalisation, and to analyse such preferences in light of holistic communication. Such knowledge can inform the development of family-centred education of healthcare personnel (HCP) on effective ward round communication for patient outcomes and patient safety.

## Methods

### Study design

Exploring the communication preferences of older patients living with frailty and of ICs during the patients’ hospitalisation required a qualitative study design that involved semi-structured individual interviews with such patients and ICs [12]. A phenomenological approach was also used to explore and interpret lived experiences of ward round communication [13]. This study was conducted according to the COnsolidated criteria for REporting Qualitative research (COREQ) guidelines for reporting qualitative research studies (see Appendix 1) [14]. The interview guide was developed in collaboration with elderly councils in the municipalities of Randers and Aarhus in Denmark to ensure that it would adhere not only to the scientific literature but also to the viewpoints of such elderly councils [15]. Two pilot interviews were conducted, after which the interview guide was slightly modified. The final interview guide is presented in Appendix 2. The principal investigator, LA, conducted all the interviews.

### Danish healthcare system

Individuals registered in the Danish Civil Registration System and whose place of residence is Denmark are permitted to access all public healthcare services in the country, including hospital admittance [16]. Healthcare coverage is tax-based. General practitioners function as the gatekeepers to these services in hospitals, unless the patient is admitted due to an acute condition, via an emergency call.

### Recruitment of study participants

Convenience sampling was used to identify potential patient and IC interviewees for this study. Patients were recruited from inpatients in the Geriatric Department of Aarhus University Hospital, an 850-bed university teaching hospital, and in the Medical Department of Randers Regional Hospital, a 191-bed regional teaching hospital. Both hospitals are in the Central Denmark Region and are city-based hospitals serving both urban and rural populations. The patient inclusion criteria were as follows: (1) 65 years of age or older, (2) suffering from frailty according to the Clinical Frailty Scale, with a score of 5–8 [17, 18] and (3) able to give their informed consent to participate in this study. ICs of inpatients in the aforementioned departments of the two hospitals were also recruited for this study. They were either contacted by phone after the patients gave their informed consent to their ICs’ participation in this study, or approached face to face if they were at the hospital. Both the patients and the ICs were briefed on this study’s purpose and methods orally and in writing, after which they were given time to consider if they would participate in this study.

### Data collection

The interviews were conducted from November 2022 to June 2023. The patients were interviewed in the hospital, and the ICs were interviewed in the hospital, on the phone or at their home, whichever they found convenient. The interviews were audio-recorded, transcribed verbatim and anonymised for subsequent analysis.

### Data analysis

Reflexive thematic analysis was employed to identify key themes from the interview responses, using an inductive coding process [19]. The six-step process used was aimed at generating thematic patterns across the dataset based on the study’s aim [20]. To ensure coding quality, RDJ, MS and KK, who are all experienced qualitative researchers, worked with LA to code the first four interviews. LA coded the remaining interviews independently, and LA and RDJ refined the themes iteratively. When information redundancy occurred, no more interviews were conducted [21]. Data analysis was performed using NVivo 14.0 (Lumivero) [22].

### Ethics

This study was approved by the Institutional Review Board of Aarhus University, Aarhus, Denmark (2023–002). All the interviewees gave their oral and written informed consent to participate in this study.

## Results

A total of 30 interviews were conducted, equally divided between older patients living with frailty and ICs. Information on the study participants can be found in Table 1. The median interview length (minimum and maximum range) with each of the patients was 32 min (18–47 min), and with each of the ICs, 40 min (26–87 min).

**Table 1:** Participant characteristics, *n* = 30

Characteristics	*n* (%) or median (range)	
	Patients	Informal caregivers
Study participants	15	15
Age, years	85 (75–100)	59 (49–77)
Gender		
Male	8 (53%)	2 (13%)
Female	7 (47%)	13 (87%)
Residency		
House/apartment	13 (87%)	–
Senior housing	1 (7%)	–
Nursing home	1 (7%)	–
Receives homecare	14 (93%)	–
CFS	6 (5–8)	–
Inpatient hospital admissions during the last 24 months	3 (1–11)	–
Relationship with patient		
Partner	–	2 (13%)
Son/daughter	–	11 (73%)
Other family	–	2 (13%)
Place of interview		
Hospital	15 (100%)	1 (7%)
At home	–	3 (20%)
Over the telephone	–	11 (73%)

*CFS* clinical frailty scale

### Building relationships and conveying information

In the context of ward rounds, the patients highlighted that their doctors sought not only to simply convey information to them but also to build a trusting relationship with them. This underscores how doctors focusing on fostering relationships can make patients feel comfortable. However, the ICs emphasised the conveyed information and their feeling of being heard rather than the interpersonal relationships. As such, information to ICs could be effectively delivered by the doctor or a nurse who is familiar with the patient.

IC 14/Daughter: ‘It’s just additional information that one might desire. It shouldn’t just go through [the patient]. It would have been nice if there had been someone else [other than the doctor] to inform us. But I know it’s a busy department. [...] Someone should have informed us when we were visiting’.

The patients often highlighted the critical role of ICs, especially spouses, when doctors convey information regarding patient care. For most of the patients, ICs are essential resources and translators between them and HCP to help them understand and remember the HCP’s messages during ward rounds. The patients expected some ICs to know everything about their medical history, even the information that they withheld. The ICs speculated that patients withheld information either because they wanted to keep it private, or they did not want to be a nuisance. Other patients were very explicit about using ICs to speak up and challenge doctors’ treatment plans.

Patient 12: ‘Now, I’m probably not the one who makes the most fuss but thank God I have a son who can make fuss for me, and he does that well’.

Both the patients and ICs highlighted the importance of aligning the level of information with patients’ preferences and current conditions. They added that this often necessitates understanding patients’ resources outside the hospital setting. Some ICs mentioned that the information they provided at the patient’s hospital admission was essential, as patients were sometimes unable to communicate due to fatigue or delirium. Consequently, ICs felt it was important for doctors to engage in conversations with them and be available for information exchange. This IC perspective, which might differ somewhat from that of the patients, was considered essential for providing a complete picture of the patient’s needs. This was rooted in their sense of responsibility, because most patients, regardless of their cognitive state, struggled to communicate their care plans to their ICs with sufficient details. The ICs emphasised that doctors need skills in incorporating ICs’ perspectives in their patient care plans and in explicitly using IC-provided information.

IC 4/Daughter: ‘I was listened to, and what I said was used in the short summary that the doctor made. My knowledge was utilised, and I couldn’t be more satisfied. I wasn’t excluded’.

The patients largely valued doctors who formed an alliance with them and emphasised mutual goals for the patient’s treatment and well-being. Many of the patients utilised terms such as ‘we’ or ‘us’ when referring to factors that affected their well-being, underscoring the importance of doctors’ prioritisation of the establishment of a patient–doctor relationship.

Patient 7: ‘He [the doctor] was down to earth and could explain what was happening. Even though he was not a craftsman like me, we had a good conversation and were able to discuss things’.

Some of the patients expressed feelings of alienation when doctors failed to perceive them as individuals, which also tended to incite frustration and anger among the ICs. For example, Patient 3 commented, ‘Even if I can’t see properly, they can still talk to me, and I can answer. They forget that you are a human being’. Likewise, according to most of the patients and ICs, doctors should be aware of patients’ feelings of being subject to a system that they may not comprehend or that they may feel subordinate to. Patient 6 said, ‘Sometimes I try to speak up, but it doesn’t always help. After all, they are the ones who are right, not me’.

Conversations about existential topics, such as attitudes towards do-not-attempt-cardiopulmonary-resuscitation (DNACPR) decisions, seemed less stressful for most of the patients. In addition, some patients noted that discussing death and other existential topics was challenging for their informal caregivers (ICs). However, this was not mentioned by the ICs. On the other hand, the ICs empathetically stated that based on their own experiences, honesty is their preferred means of communicating bad news or handling advanced directives, including from doctors. Most of the ICs preferred that the doctor initiate this talk.

IC 5/Partner: ‘You get to know what it [the cancer diagnosis] is all about. What do we do from here, what is the prognosis, what are the long-term consequences. Simply facts. In a kind, matter-of-fact and compassionate way, without it turning into hugs and tears’.

As for surrogate decisions for DNACPR decisions, the ICs emphasised that the doctor should explicitly explore ICs’ knowledge of patients’ preferences, as this would minimise the IC’s strain. Furthermore, most of the patients considered the burden on the IC after the patient’s resuscitation and did not want to be a nuisance, which may have led some patients to decline resuscitation attempts.

Patient 11: ‘But the second time [I was asked about my DNACPR decision], I said I only wanted to be kept alive if there is a meaning to it. [...] And another thing is that [my spouse] should not have the responsibility of getting me into a nursing home’.

### Alleviating informal caregiver (IC) strain

The ICs frequently conveyed significant levels of burden and concerns, notably highlighting their challenges associated with the discharge process. This encompassed managing home care responsibilities and ensuring that the patient’s residence was adequately prepared, often while experiencing the additional strain of attending to other family members’ needs.

IC 12/Daughter: ‘So, what I’ve felt pressured and stressed about is that I’ve kind of felt like it was all on me; that I had to bear my mother’s stress over this situation. [...] So, I’ve felt like I’m the one who’s had to hold it all together and then accommodate other people’s frustration’.

Although the patients relied heavily on the support of their ICs during their hospitalisation, they often hesitated to impose burdens on their ICs, reflecting their reluctance to be a nuisance. Additionally, they frequently affirmed their fundamental trust in medical authorities, as exemplified by their acceptance of doctors unilaterally determining their care plan without seeking their informed consent.

Patient 5: ‘No [I wasn’t asked about a treatment], but I reckon it’s all fine. […] I’m entirely comfortable with that’.

On the other hand, the ICs often shared their encounters with an overburdened healthcare system—a system that demonstrated minimal compliance with directives pertaining to patient care during a patient’s hospital admission. Furthermore, the ICs often felt deprioritised by HCP due to their lack of information on patient care.

IC 14/Daughter: ‘Sometimes [we] don’t get prioritised at all. [...] I think it’s been frustrating, especially when [the patient] was hospitalised and he felt really bad’.

Navigating their lack of trust in the healthcare system and, consequently, also in doctors, was a source of stress for most of the ICs. They stated that this issue of lack of trust could be alleviated through doctors’ demonstration of genuine interest in their patient’s story and their good preparation.

IC 10/Daughter: ‘They actually knew what was in her medical record. They knew what it was about when they showed up at the ward. They had read everything that had happened before. So, we felt completely safe, and a weight was lifted off my shoulders’.

Most of the ICs stressed their role as an advocate and their responsibility to ensure quality of care for their patient, as illustrated by IC 12/Daughter, who stated: ‘And then, I can just see the course of illness he [the patient] has had. Well, he would have been sent home without follow-up if I didn’t do anything. […] So, I feel like I have to double-check all the time’. However, when time was limited, the ICs prioritised their patients’ needs, which might have conflicted with their desire for active participation during ward rounds. In addition, assuming primary responsibility for a hospitalised patient’s care plan while feeling excluded from crucial information posed significant challenges for many of the ICs in their supportive role.

IC/Daughter 10: ‘Had we not been there, she would not have been able to get help [from the ward]. It was tough’.

Both the patients and the ICs noted that doctors’ demonstration of ample time and patience was of great importance to the patients. The patients typically valued the time doctors spent with them, focusing more on the amount of time given rather than the quality of information shared during ward rounds. On the other hand, the ICs sought dedicated time from doctors and comprehensive information sharing, including for the ICs’ sharing of their version of the story, often referred to as ‘the long story’.

IC 12/Daughter: ‘There was time enough for the doctor to take the whole story. He didn’t just focus on the reason for this hospitalisation, and that was nice’.

Many of the ICs stated that their patients’ frailty had led to their frequent hospitalisation and to subsequent changes in their functional abilities and care needs. Therefore, when the ICs were given the opportunity and time to tell ‘the long story’, their burden decreased.

### Sharing the decision-making process

The patients’ decision-making preferences were found to encompass a spectrum. While most patients preferred their doctor or IC to decide on their care plan, a few of them preferred to make their own care decisions. This seemed based on their respect for medical authorities.

Patient 1: ‘It’s the doctor and I [who make the decision]. And here, I know who is the smartest. […] that’s why I don’t speak up’.

The patients who preferred to be involved in the decision-making process needed to be actively involved in it. Consequently, this was fundamentally the doctor’s responsibility. For example, Patient 11 stated, ‘What I’m trying to say [is] … I’m trying to get that through to the doctor […]. It is imperative that [the] doctor includes me’. One area in which both the patients and the ICs did not feel included in the decision-making process was regarding the patients’ hospital discharge. For some of the patients, this resulted in their lower compliance with their medical treatment.

Patient 6: ‘That’s probably the problem—that doctors make decisions on my behalf [...] but even if I find it difficult, I do as I please anyway [after my discharge]’.

The patients and the ICs recommended that for them to be part of this decision-making process, doctors should provide patients with few and easily understandable options. The ICs appreciated prompt and comprehensible information, considering their needs, as the discharge process constituted a major burden for them. One strategy for lightening the IC burden was to align expectations and clarify goals to be met before the discharge. However, as some of the ICs mentioned, to some extent, they have an influence on the decisions. IC 4/Daughter said, ‘But sometimes, [when she makes decisions about patient care], I nudge her a little. Shouldn’t you…? Have you thought about …? We do that together when I call her’.

The patients’ inclination to build relationships with their doctors led many of them to express a preference for equal communication with their doctors. On the other hand, many of the ICs, said they could not say what was on their mind in front of the patient, as when they disagreed with the patient, maintaining a good relationship between them was essential. Thus, the ICs valued an opportunity for them to talk with the doctor alone.

IC 15/Daughter: ‘So, they asked me if there was anything I wanted to add. Yes, there was a lot, but not while my father was present. I have often found someone out in the hallway and asked that person to request the doctor to call me’.

Some of the ICs noted that doctors sought information or sought to establish an alliance with them that could influence patients to behave in a certain manner, which the staff found advantageous. Likewise, some of the ICs observed that doctors primarily requested their involvement when the patient did not comply with the staff directives. This was particularly evident when the patient had cognitive disabilities, was reluctant to eat or drink sufficiently or neglected their illness. However, as a few patients mentioned, their ICs were sometimes mistaken or overly protective of them.

## Discussion

This qualitative analysis of interviews with older inpatients with frailty and ICs identified three themes that encompassed their similar and diverse communication perspectives and needs with regard to ward rounds.

### Building relationships and conveying information

The patients emphasised the importance of fostering equal relationships with doctors. This result possibly reflects generational shifts in behaviour, as previous research has suggested that older patients tended to defer more to medical authorities [23]. Fostering equal relationships with patients (i.e., by cultivating interpersonal skills) is embedded in pre- and post-graduate medical education curricula, such as via the CanMEDS role of the Communicator of ‘Establish[ing] professional therapeutic relationships with patients and their families’, and plays a dominant part in, for example, nurses’ education [24, 25].

The patients added that doctors should not only demonstrate empathy for their patients but should also convey patience with them and a sense of having ample time for them as markers of quality care, which should thus be emphasised in their ward-round communication skills training. Similarly, the patients underlined the importance of doctors’ relationship-building skills given the time constraints prevalent in hospital settings, characterised by short admissions, lack of service continuity and busy staff [26]. Nevertheless, as in previous studies, some of the patients in this study were objectified and treated more as tasks to be managed rather than as human beings [27]. Today’s healthcare communication with older patients often reflects unequal power dynamics and reinforces stereotypes of frailty and dependency, which is often described as ageism [28, 29]. Implicit stereotyping of older patients in doctors’ interactions with them can affect these patients’ perceptions and health outcomes [30]. The current study reiterates how condescending behaviour of doctors impacts both patients and their ICs.

We also explored the participants’ experiences with DNACPR decisions, as these decisions often occur during ward rounds. Previous studies have found that DNACPR decisions challenge doctors due to the ethical, emotional and legal complexities involved [31, 32]. In the current study, most of the patients did not express discomfort when speaking to their doctors about existential topics, such as death or DNACPR decisions. They regarded death and dying as inevitable and beyond their control. Lloyd et al. (2016) also explored the experiences of older adults living with frailty and approaching death, and the experiences of their ICs, to understand their multidimensional needs and how a palliative approach might be relevant for them [33]. The researchers argued for addressing the subjects’ future concerns rather than centring the conversation with them on death. Interestingly, some of the patients in Lloyd et al.’s study noted that their ICs might feel uncomfortable discussing death, a sentiment that contrasted with the ICs of this study’s statements. This claim of the patients might have been rooted in their reluctance to be a nuisance to their relatives and to cause them pain [34].

Most of the ICs preferred honesty when discussing DNACPR status with doctors, as found in previous studies [35]. Educating doctors in handling DNACPR decisions is a multifaceted task, oftentimes solely focusing on clinical decision-making skills, rather than, for example, communication or psychological support training [36]. Here, bringing in patients or ICs as educators or evaluators of this task could be advantageous. Similarly, Sivertsen and colleagues found that insufficient communication about the DNACPR decision-making process can lead to distress or feelings of powerlessness among ICs [35]. Furthermore, this could cause strained relationships with healthcare providers and potential long-term psychological effects [35].

### Alleviating IC strain

Strain among ICs has been demonstrated in several studies [37–39]. A study in Switzerland found that ICs’ feeling of unpreparedness caused their sense of burden [37]. In the current study, however, the ICs almost unanimously reported that the primary source of their strain was their feeling of being responsible for patient care. In Denmark, ICs are often not involved in home care but, instead, manage the patient’s transition from the hospital to the home with all relevant stakeholders. Thus, as shown in previous research, ICs’ exclusion from the discharge process increases their burden [38]. IC strain may also arise from trust issues. In the present study, we found that younger ICs generally distrusted doctors and the healthcare system more than did older ICs. As previous research indicated, this can lead to severe IC discomfort [39]. Moreover, a Danish study in 2018 explored the collaboration experiences of relatives of older patients with hospital personnel and their involvement in the patients’ care and treatment [35]. Similar to the current study, the relatives in that study felt an absence of care and, thus, felt the responsibility for patient care placed on them [35]. In addition, in the current study, the ICs experienced difficulties with being informed during ward rounds. Riffin et al. (2020) highlighted that while such integration of ICs in patient care could help improve patient care, HCP’s lack of time to do so is a major barrier [40].

### Sharing the decision-making process

In the current study, the patients’ preferences regarding their participation in the decision-making for their care varied, so doctors should explicitly ask their patients if they wish to participate in such decision-making [23]. Ekdahl et al. (2010) highlighted how older patients, particularly those living with frailty, juxtaposed their desire to be informed about the decision-making process with their desire to participate in such process [41]. However, in the current study, we did not find a strong inclination towards the belief that being well-informed necessarily translates into active involvement in decision-making. This may be because most of the patients did not perceive themselves as part of the decision-making process or chose not to participate in it. Similarly, Bastiaens et al. (2013) found that many patients among older community-dwelling people in Europe desired to be involved in their own care, but ‘their definition of involvement [was] more focused on “[a] caring relationship”, “[a] person-centred approach” and “receiving information” than on “active participation in decision making”’ [42]. The ‘caring relationship’ between patients and doctors was an important factor of the patients’ feeling of control and well-being, as Nyende et al. (2023) [43].

In the context of the discharge process, where ICs’ knowledge is particularly relevant, previous studies indicated a deficiency in shared decision-making practices [38]. If patients and ICs are to be parts of this process, doctors should provide few and easily understandable options to patients. In the current study, when the ICs were invited to join the discharge process, both the patients and they benefitted significantly. Other studies have demonstrated that involving patients and ICs in the shared-decision process regarding hospital discharge enhances care plan efficacy and satisfaction with subsequent arrangements [44].

Finally, we found that ICs may find themselves navigating the complex and changing aspects of paternalism, when the alliance between the doctor and the IC appears to be prioritised over the patient’s autonomy. When paternalism is warranted, it requires ICs to collaborate with doctors to foster an alliance that values the patient’s voice and preferences as central elements in the decision-making process.

## Limitations

The current study had some limitations. First, we managed to have only one patient and one IC who were related. If we had interviewed more patients and ICs who were related, our analysis would have been deeper, as related patients and ICs might have different perspectives from unrelated ones. However, the mostly unrelated patients and ICs in the current study might have been more honest in their interview responses. Second, we were unable to include more than two male ICs, suggesting that most primary ICs are female [45]. This predominance of female over male ICs in our interviews could have affected our analysis, as female ICs have been shown to express more IC burdens than male ICs [46]. Third, as this study was a qualitative study conducted in Denmark, our findings were shaped by cultural factors, including healthcare systems and family structure. Fourth, we interviewed the patients during their hospital stays, which resulted in relatively short interviews. However, we decided to interview patients while they were still hospitalised, because previous studies in comparable contexts had challenges in recruiting patients after their hospital discharge [47].

## Conclusion

This qualitative study explored, through interviews, the perspectives of older patients living with frailty and of ICs on ward-round communication. Our analysis generated three themes that covered the perspectives and needs of patients and ICs and highlighted the importance of interpersonal communication between doctors and patients while maintaining a collaborative approach to ICs. These themes are the reliance of the patients in this study on both doctors and ICs for making care decisions, and the ICs’ valuing of the recognition of their narratives and burdens and of their inclusion in the decision-making process. These themes emphasise the necessity of tailoring communication to address the unique needs and circumstances of each patient and their IC. This holistic approach could lead to more effective and patient-centred care that will enhance overall patient satisfaction and alleviate IC burden.

## Supplementary Information

Below is the link to the electronic supplementary material.

Supplementary file1 (DOCX 24 KB)

Supplementary file2 (DOCX 31 KB)

## Data Availability

The data are not available for deposition in a public repository.
