# Supplementary material for: “They forget that I’m a human being”—ward round communication with older patients living with frailty and informal caregivers: a qualitative study
Source: Eur Geriatr Med. 2024 Sep 4;15(5):1383–92. doi: 10.1007/s41999-024-01043-5 (PMC11614924; doi:10.1007/s41999-024-01043-5)
Supplement: Supplementary file 1 — Supplementary file1 (DOCX 24 KB) [file 41999_2024_1043_MOESM1_ESM.docx]

**APPENDIX 1**

**Consolidated criteria for reporting qualitative studies (COREQ): 32-item checklist**

Developed from:

Tong A, Sainsbury P, Craig J. Consolidated criteria for reporting qualitative research (COREQ): a 32-item checklist for interviews and focus groups. *International Journal for Quality in Health Care*. 2007. Volume 19, Number 6: pp. 349 – 357

| Item no. | Guide questions/  description |  | Reported on Page # |  |
| --- | --- | --- | --- | --- |
| Domain 1: Research team and reﬂexivity | | | |  |
| *Personal Characteristics* | | | |  |
| 1. Interviewer/  facilitator | Which author/s conducted the interview or focus group? |  | Page 4 / paragraph 1 (line 8) |  |
| 2. Credentials | What were the researcher’s credentials? E.g. PhD, MD |  | MD |  |
| 3. Occupation | What was their occupation at the time of the study? |  | Full time PhD student, Geriatric Resident |  |
| 4. Gender | Was the researcher male or female? |  | Female |  |
| 5. Experience and training | What experience or training did the researcher have? |  | Master classes in qualitative research studies, interviewing patients during inpatient visits |  |
| *Relationship with participants* | | | |  |
| 6. Relationship established | Was a relationship established prior to study commencement? |  | No relationship between the principal investigator and patients or informal caregivers had been established before the study began |  |
| 7. Participant knowledge of the interviewer | What did the participants know about the researcher? e.g. personal goals, reasons for doing the research |  | Patients were informed about the goal of the overall project; to improve ward round communication from the patients’ and informal caregivers’ perspective. |  |
| 8. Interviewer characteristics | What characteristics were reported about the inter viewer/facilitator? e.g. Bias, assumptions, reasons and interests in the research topic |  | As a geriatric resident, the principal investigator were familiar with the ward round setting, and having general interest in communication and educational training for years. |  |
| **Domain 2: study design** |  | |  | |
| *Theoretical framework* |  | |  | |
| 9. Methodological orientation and Theory | What methodological orientation was stated to underpin the study? e.g. grounded theory, discourse analysis, ethnography, phenomenology, content analysis | | Page 4 / paragraph 5 (line 2) | |
| *Participant selection* |  | |  | |
| 10. Sampling | How were participants selected? e.g. purposive, convenience, consecutive, snowball | | Page 5 / paragraph 4 (line 1) | |
| 11. Method of approach | How were participants approached? e.g. face-to-face, telephone, mail, email | | Page 5 / paragraph 2 and 3 | |
| 12. Sample size | How many participants were in the study? | | Page 7 / paragraph 1 (line 1) | |
| 13. Non-participation | How many people refused to participate or dropped out? Reasons? | | No data available. Fatigue were the predominant reason why patients refused to participate, while informal caregivers, especially spouses, mainly decline participation because they were overwhelmed by an excessive informal caregiver workload | |
| *Setting* |  | |  | |
| 14. Setting of data collection | Where was the data collected? e.g. home, clinic, workplace | | Page 6 / Table 1 | |
| 15. Presence of non-participants | Was anyone else present besides the participants and researchers? | | No | |
| 16. Description of sample | What are the important characteristics of the sample? e.g. demographic data, date | | Page 6 / Table 1 | |
| *Data collection* |  | |  | |
| 17. Interview guide | Were questions, prompts, guides provided by the authors? Was it pilot tested? | | Page 4 / paragraph 5 (line 4-7) | |
| 18. Repeat interviews | Were repeat interviews carried out? If yes, how many? | | No | |
| 19. Audio/visual recording | Did the research use audio or visual recording to collect the data? | | Page 5 / paragraph 4 (line 4) | |
| 20. Field notes | Were ﬁeld notes made during and/or after the interview or focus group? | | Yes | |
| 21. Duration | What was the duration of the interviews or focus group? | | Page 7 / paragraph 1 (line 2-4) | |
| 22. Data saturation | Was data saturation discussed? | | Page 5 / paragraph 5 (line 4-5) | |
| 23. Transcripts returned | Were transcripts returned to participants for comment and/or correction? | | No | |
| **Domain 3: analysis and ﬁndings** |  | |  | |
| *Data analysis* |  | |  | |
| 24. Number of data coders | How many data coders coded the data? | | Page 5 / paragraph 5 (line 2-4) | |
| 25. Description of the coding tree | Did authors provide a description of the coding tree? | | No | |
| 26. Derivation of themes | Were themes identiﬁed in advance or derived from the data? | | Derived from data. | |
| 27. Software | What software, if applicable, was used to manage the data? | | Page 5 / paragraph 5 (line 5-6) | |
| 28. Participant checking | Did participants provide feedback on the ﬁndings? | | No | |
| *Reporting* |  | |  | |
| 29. Quotations presented | Were participant quotations presented to illustrate the themes/ﬁndings? Was each quotation identiﬁed? e.g. participant number | | Yes | |
| 30. Data and ﬁndings consistent | Was there consistency between the data presented and the ﬁndings? | | Yes | |
| 31. Clarity of major themes | Were major themes clearly presented in the ﬁndings? | | Yes | |
| 32. Clarity of minor themes | Is there a description of diverse cases or discussion of minor themes? | | Mentioning of “most patients” or “some patients” | |
