# Supplementary material for: “They forget that I’m a human being”—ward round communication with older patients living with frailty and informal caregivers: a qualitative study
Source: Eur Geriatr Med. 2024 Sep 4;15(5):1383–92. doi: 10.1007/s41999-024-01043-5 (PMC11614924; doi:10.1007/s41999-024-01043-5)
Supplement: Supplementary file 2 — Supplementary file2 (DOCX 31 KB) [file 41999_2024_1043_MOESM2_ESM.docx]

Appendix 2

Interview guides

| **Patients** | | |
| --- | --- | --- |
| **Theme** | **Questions** | **Further questions** |
| Context | How are you today?  How would you describe your current health?  Try to describe your expectations of being hospitalized | Are you ready for our talk?  Why are you hospitalized? Have you previously been hospitalized and where?  Why were you hospitalized and who referred you? The family, the doctor, sudden illness?  How did you experience being hospitalized? |
| Understanding the given information at ward rounds | Try to remember a conversation in the hospital where you were a patient, and the doctor had to tell you somthing about your treatment. It may be during this or your previous hospitalizations.  Try telling me something about that conversation?  How did you experience talking to doctors and nurses at the interview?  What will make it easier or harder for you to understand what the staff is saying at the interview?  When the conversation is finished, do you feel able to convey to your relatives what was talked about and what decisions were made? | What made it a good or bad conversation? |
| Framing ward rounds (DPC: The doctor-patient conversation - use what the patient understands) | What comes to mind when I say "ward rounds"?  What expectations do you have for such a conversation/ward round?? | Do you know what you typically talk about during a ward round/DPC (content)?  Do you know what the purpose of the ward round/DPC is? |
| Involvement in the decision-making process | Now we will talk about how decisions are made in the hospital.  Try to think about some of the examinations and treatments that you have had done here at the hospital - it may be during current hospitalization or earlier.  Do you feel that you are involved in the decisions that doctors make about examinations, treatment or medication, etc.?  What thoughts have you had about your preferences for treatment decisions?  Try to think about the last time you had ward rounds and decisions were made about, for example, changing medication or circumstances surrounding your discharge. What were you talking about during that round?  What decisions had to be made about?  How did you experience being involved in the decision?  Should you decide on something?  When looking at other studies, it is quite different whether older patients want to decide about their treatment while they are hospitalized or whether they prefer for the doctor to make treatment decisions.  Why do you think it can be so? | Are you being informed well enough about what is happening?  Follow-up: What was it that made you involved or didn't get involved?  How important is it for you to be able to decide for yourself?  What happens with decisions when you are admitted to hospital? And why?  What expectations do you have of being involved in decisions? (Satisfaction)  Who are your advisors on this? Is it the doctors you rely on?  Did you make any decisions?  Do you find that the staff knows your attitude to deciding what should happen when you are hospitalized?  How do the staff best ask about your attitude to wanting to decide during hospitalization? |
| Patient satisfaction | [located under other questions] | What was it that made you satisfied with the conversation? |
| Difficult topics for ward rounds | Now we are going to talk about something that can be difficult for some to talk about, both patients and staff. And if it is difficult for you, you do not need to answer the following questions.  Have you ever experienced that you had to receive notification of something bad, e.g. about cancer or the like?  How did you experience that conversation?  It's never nice to be told that you're failing something bad, but how do you think you'd rather be told about, for example, a scan of a lump that may be cancerous? | What was good or bad in the conversation you had?  In your opinion, how can the doctor best convey a difficult message to you? |
| Death and dying | Another thing that is also part of life is that we have to leave here sometime. It is different how and how much families talk about death.  How do you talk in your family about the end times and have you talked about death in your family?  At the hospital, the doctor decides whether a patient with cardiac arrest should be resuscitated or whether life should take its course. It is therefore the doctor's responsibility, unless the patient has previously made up his or her own mind.  Have you been asked about your opinion on possible cardiac arrest resuscitation attempts?  How would you like to be asked about your opinion on cardiac arrest resuscitation?  What do you think about getting involved in the decision of resuscitation?  How do you feel about your relatives being involved in the decision to attempt cardiac arrest? | Is it a difficult topic for you to talk about? (taboo?) |
| Closure | Is there anything else that comes to mind when we talk about how you and the staff talk to eachother during rounds?  Do you or any other questions or comments?  May I contact you again if I get home and see that I forgot something? |  |

| **Informal caregivers** | | |
| --- | --- | --- |
| **Theme** | **Questions** | **Further questions** |
| Context | Try to tell me something about yourself and your relationship with your loved one/(NAME)/(relationsship with patient)?  Try to tell me somthing about what you know about your loved one's illnesses?  How much can you know about your relative's hospitalization for your loved one?  How much may you be allowed to decide for your relative?  What would you like to help your loved one with during hospitalization?  Why do you want to help your loved one while she is hospitalized? | How often do you see each other?  Do you assist with cleaning, grocery shopping etc.?  Do you know why he/she is hospitalized?  Did you and your relative agree upon the extend that you may be involved and how?    What motivates you? |
| Framing ward rounds (DPC: The doctor-patient conversation - use what the patient understands) | What do you understand when I talk about ward rounds?  Do you have a feeling that your loved one understands what the ward rounds are all about?  How should the doctor talk to you so that you understand how your loved one is doing during hospitalization?  Do you have a feeling that your loved one understands what doctors/caregivers tell them during ward rounds? | Or can remember details from the ward round? |
| Participation in decisions (only if the relative has been mandated to be involved according to context) | Now I would like to talk about how informal careivers are best involved when the older patient is admitted.  Think about the last time your loved one was hospitalized. When was it and where? Why was your relative hospitalized?  How did you experience the last ward rounds with the doctor/nurse?  How did you experience being involved in decisions made for rounds?  Did you experience being involved in decisions in relation to, for example, medical treatment and investigation, medicine?  How did you experience being involved in discharge decisions? | Have you been contacted after ward rounds by a doctor or nurse or were you present?  Try to describe how you experienced your role as a relative during rounds to an inpatient?  What areas could your relative and you disagree on? |
| Difficult topics for ward rounds | Now we are going to talk about something that can be difficult for some to talk about, both for patients and staff. And if it is difficult for you, you do not need to answer the following questions.  Have you ever had a doctor talk to you about resuscitation in case of cardiac arrest or whether your relative needed to be put on a ventilator?  How did you experience this conversation? | What made this conversation good or bad? |
| Surrogate decision-making | In the United States, informal caregivers in up to half of older people's admissions experience that the older person is so weakened or confused that he/she cannot make decisions or express his/her opinion.  Have you experienced having to make decisions about treatment or the like because your relative was not able to talk to the doctor himself?  Sometimes patients are not able to communicate, because they are seriously ill. Do you know your relative's attitude to, for example, resuscitation or other life-prolonging treatment?  Have you and your relative agreed whether you have a mandate to comment on your relative's position on resuscitation, etc.? |  |
| Trust and its prerequisites | Now let's talk about trust in healthcare professionals.  Try telling me something about one of the experiences where you experienced having good trust or poor trust in the staff. | What made this experience good or bad? How were your feelings met? |
| Closure | Is there anything else that comes to mind when we talk about how you and the staff talk to each other during rounds?  Do you have any other questions or comments?  May I contact you again if I come upon something I forgot to ask you? |  |
